# On the Tests of Death

**Published:** 1850-04

**Authors:** 


*On the Tests of Death.* By Bennet Dowler, M. D., of New Orleans.

We select the following from an essay entitled "*Researches on the Natural History of Death. By Bennet Dowler, M. D., etc., of New Orleans,*" published in the New Orleans Medical and Surgical Journal for March, 1850, and also issued in a pamphlet form. The essay displays independence and originality of investigation, as do all the contributions emanating from the pen of this distinguished observer; and it is written in the sprightly, racy style which characterizes his composition. We should be glad, if we had space, to transfer to our columns the entire essay. We have selected the portion relating to the criteria of the certainty of death, containing the criticisms of the author upon the memoir upon this subject by M. Bouchut, which received, from the Academy of Sciences of Paris, the award of the Manni prize. A translation of this memoir, by Prof. Bryan, was presented to our readers in a former number of this journal. [See vol. IV, page 463]—Ed. Buf. Med. Jour.

I return from this digression to the subject of the Manni prize, which was placed, by a royal decree, at the disposal of the French Academy in 1837. The subject was important. The public had a natural aversion to living entombment. How to avoid this was the great question. The philosophers, as well as other people, without hesitancy, admitted that putrefaction was a certain test of death, but in nothing else could they agree. The prize of the Abbe Manni was founded with hope of discovering some other satisfactory sign *anterior to that of decomposition*, for the inconvenient delays, and the deleterious effects of keeping the dead until this repulsive change had taken place, were obvious, and were the very things to be anticipated and prevented.

Besides, in some climates,* the test of putrefaction would not occur for weeks or months, unless the rooms be artificially heated to a high temperature, which, to the poor, would be both inconvenient and expensive, and often impracticable during epidemics, and, in cases of contagious diseases, unsafe. The prize question did not contemplate this *test at all*, seeing that it comes too late.

It is conceded that the award of the Academy was made conscientiously; that the paper crowned with the prize, was the best among the multitude offered; and that the Academicans are, as a body, unsurpassed for general learning and science. But the Academy has no monopoly of nature, nor of her true interpretations. Her platform of facts and her voice of truth are alike accessible to all mankind; and her best Academicans are those who read her volumes most correctly.

According to M. Bouchut, the successful candidate for the prize, the "certain signs of death are *immediate or remote.* The first consists in: 1, the prolonged absence of the sounds of the heart; 2, the simultaneous relaxation of the sphincters; and, 3, the sinking of the globe of the eye, with loss of transparency of the cornea. The first of these, alone, is regarded by the committee as conclusive. The *remote* signs are; 1, cadaveric rigidity; 2, the absence of muscular contractility under the influence of galvanism; and, 3, putrefaction."

The first cannot be a very general test. For, passing by this test as *satisfactory in itself*, its application must be limited, because there are but

* In Russia, during the long winter, the animals killed for food are preserved wholly by the climate, no salt or other antiseptic being needed. The meat stowed away or hung up in market, is frozen at once, and continues so till spring.

few good stethoscopists among good practitioners, and, in the best hands, certainly is often not attainable.

I speak not of the ideal, but of the actual state of ausculation. In some cases where able ausultators have pronounced the heart altered or diseased, I have found it healthy on dissection. This is one difficulty. But this is not the worst. The Academy, and M. Bouchut, take for *granted*, that which may not be *true*, *and which is the very thing to be proved!* Who has proved that the heart, like the pulse, like every other organ, may not fall into *temporary quiescence or inaction?* May not the heart itself suffer apparent, not real death, as all analogies drawn from other muscular organs teach? The sphincters, uterus, intestines, and stomach, the respiratory, lingual, occular, and locomotive muscles may be palsied, inactive, apparently dead for a time. It is a downright begging of the question, to assume that the heart *cannot itself fall into this very state of apparent death.* The natural history of the movements of the heart, indicates the probability of a temporary suspension of action; at one time it gives 200, at another eight or ten strokes in a minute; it intermits, or is irregular. In cholera it is probably cramped in some cases; temporarily quiescent in others. Moreover, it has, in common with other muscles, a kind of life of its *own;* it is not the known sole criterion of general life. Comparative physiology shows that an animal may live hours without the heart, and the heart for days* without the body. An alligator's heart will act with regularity for many hours, perhaps for days, after having been cut out of the body, and emptied of its blood. Let an alligator thus deprived of its heart, be roasted; return its heart, and apply the stethoscope, and then the dead will aford this certain sign of life! The commission of the Academy cannot object to this argument, because they themselves experimented on the inferior animals in testing M. Bouchut's claims: as for myself, I have shown, in my published papers, that I attach less importance to comparative physiology, as the interpreter of human physiology, than systematic writers do themselves. If I take their own point of departure, they can require nothing more.

NOTE.—In the instance of a patient coming under our observation during the last summer, who died of chronic diarrhœa, no cardiac sounds could be discovered, on careful examination with the stethoscope, for several days before death took place. Our attention was directed to this case, as illustrating the fallaciousness of this test of death.—ED. BUFFALO MEDICAL JOURNAL.

The second great sign of death is, according to M. Bouchut and the Academy, the relaxation of the sphincters. When I say that this is as great a mistake as was ever uttered, I speak from an experience in this particular line, that probably has never been equalled—I can hardly imagine that any sane experimenter will take the same pains in its verification—a verification incidental to several years' experiment on animal tem-

* This I cannot vouch for as an observation made by myself, but Dr. Lindsay, of this city, has seen the separated heart of the alligator still in action, on the second day after its removal from the body, which I fully believe, though I have never watched the heart more than 6 to 9 hours continuously after separation.

perature,—a single experiment often lasting several hours, during which thermometers have been repeatedly passed within the sphincters. The latter, with very few exceptions, contract strongly after death. Relaxation of the sphincters is not a post mortem, but an ante—or præmortem phenomenon. It happens, sometimes, as a disease, and it is not a fatal symptom. I have seen the sphincter ani completely palsied, open, shapeless. Its relaxation is a frequent occurrence during the agony; but when that ceases, the tenacity of the sphincter ani, for example, returns immediately, and, what is more, after prolonged artificial dilatation with the thermometer, quickly closes, preventing the escape of large accumulations of liquids, as in cholera, yellow fever, etc. In passing through the wards of a hospital, an observer will notice among the dying, even in the visage, relaxations, which will often disappear in a few minutes after death. It is before, not after death, that the orbicular muscle is relaxed, the mouth open, the under jaw fallen, and so on.

The third and last sign relied on by M. Bouchut and the Academy, "is the sinking of the globe of the eye, and a loss of transparency of the cornea." This sign has long been much insisted on ; but it utterly fails as a uniform criterion of death, as I could show. I will only remark that this change happens before death in some cases, and often it does not appear after death in time to be useful, particularly, in some cases of death of yellow fever, the eyes remaining prominent for a considerable period, even for hours. The cornea, in many cases, can be preserved for hours in a transparent state by closing the lids, and carefully excluding the air. The experiment will be the more striking, by keeping one eye open, and the other shut, the former soon becomes dim or glassy from simple desiccation ; in the absence of natural secretion ; the latter will be nearly as clear as in health.

Does not the globe of the eye recede, and its convexity diminish before death, in many cases of cholera ! I incline to the affirmative.

The cornea may become *flattened, dry, and without transparency before death !* This fact, to me quite new, can be well authenticated, if necessary : 1849—Feb. 11 ; James Garner's child, a female, born in England, aged three—sick three days with cholera complicated with congestion of brain; insensible for twenty-four hours; cramps; severe clonic convulsions, sometimes general, sometimes local ; otherwise motionless ; takes no notice; eyelids widely open and fixed, as in staring, never winking; *cornea, dull glassy, flattened* in appearance; as dry as paper. Washes caused no winking. Feb. 12; *the desiccation of the cornea increased, flattened, lustreless,* almost horny; pupils dilated, insensible to light, fixed—a gummy mucosity exuding from the conjunctiva. The following day, the condition of the eyes was the same. The coma increasing, the child died. Here, *before death,* is an example of the very changes in the eye, so much relied on as the sign of death, with this difference, that among many hundreds of the dead, it would difficult to find a more aggravated and frightful case, anterior to putrefaction.

*The Revue des deux Mondes*, (Oct. 1848,) hails M. Bouchut's important suggestion of auscultation, as the test of death, as a discovery absolutely perfect, leaving nothing more to be desired, dissipating every fear, even in the most timid, as to the danger of premature interment, and, thereupon, glorfies France, as the benefactor of the whole human race, in these exulting words: "C'est a la France, if est bon de le rappeler en finissant, que revient le double honneur d'avoir, la premiere, pose le probleme des signes de la mort sur le terrain scientifique, comme de l'avoir, la premiere aussi, scientifiquement resolu."

I propose the thermometer as a means of testing death, possessing, as it does, superior certainty over the stethoscope. The latter method takes for granted, that in apparent death, the heart's action continues; that it cannot be for a time suspended, and that its action can always be heard! The very analogies of apparent or temporary death seem to oppose or contradict these assumptions. The analogies and the positive facts known of animal temperature, teach that, during life, the body is not heated and cooled like inert matter. Place two or three thermometers in the arm-pits—in the bend of the arm, (the fore arm being flexed,) in the mouth and within the sphincters, to ascertain the heat of the surface, and of centres, (the rectum is the best, and most accessible centre). The application of the thermometer requires no skill, and is open to the inspection of all, and is a test for all the warm blooded animals—at least for man. While the auscultatory test takes for granted that there can be no temporary inaction of the heart, and that all its motions can be heard; the thermometrical test takes nothing for granted without the most indubitable proof. Its great axiom is that man, in his living state, maintains an uniform temperature, independent of the surrounding media, while a dead man, like other inert matter, has no independence of this kind, but steadily responds to, and is governed by, calorific condition altogether physical—heating and and being heated, receiving and radiating caloric. This is not the result of speculation, but of prolonged and varied experimental research.

The refrigeration of the body before death, in cholera, congestive, and the like, is not physical refrigeration, responding to the calorific condition of the surrounding media; it is a morbid or physiological caloricity, which, for a time, augments or continues stationary after death, until it shall be replaced by physical refrigeration, as its phenomenal history clearly shows.

The facts which I have published concerning post mortem caloricity, do not invalidate this thermometrical test; for soon or late, the physical refrigeragation must take place. I may here add, that the speculative opinion which prevails among those who do not take the trouble to make experiments, namely, that these calorific movements are the effects of putrefaction, is wholly unfounded, (so far as it regards the human subject;) how much soever it may be countenanced by certain analogies derived from other inert matter. The calorific, and the putrefactive periods, so far from coinciding, antagonize each other, so long as the heat is not in accordance with the ordinary physical laws of caloric. The point of coincidence and equilibrium, is really the point of putrefaction, unless the circumstances be of an extraordinary character, such as involve the freezing point, or that of torrefaction. But the predomination of the invariable law of physical refrigeration, is a criterion always attainable, and may be proved, as to its times, distances and velocities, by arithmetical calculation: ascertain the temperatures of the media, and of the heated body; the velocity of the refrigeration will be proportioned to the times and distances, and will proceed from the surface to the centre, until the equilibrium be attained. The only objection that lies against this rule relates to calorific conditions, where the differences between the heated body and the media are very slight; but this is of no importance in practice, because there is always a marked difference between the average temperature of the air in the shade, and that of a living, or recently dead person.

